# Transtheoretical model-based mobile health application for PCOS

**DOI:** 10.1186/s12978-022-01422-w

**Published:** 2022-05-12

**Authors:** LianHong Wang, Ying Liu, Huiwen Tan, Shiming Huang

**Affiliations:** 1 Nursing Department of Affiliated Hospital of Zunyi Medical University, Zunyi, 563000 Guizhou China; 2 Nursing College of Zunyi Medical University, Zunyi, 563000 Guizhou China

**Keywords:** Polycystic ovary syndrome, Randomised control trial, Mobile health application, Transtheoretical model

## Abstract

**Background:**

Lifestyle modification (diet, exercise, and behavioral interventions) is the first-line treatment for polycystic ovary syndrome (PCOS). The benefits of face-to-face lifestyle modification intervention in a short time have been demonstrated. However, few studies have investigated the mobile technology effects on lifestyle modification in PCOS. Therefore, we examined the effect of transtheoretical model-based mobile health application intervention program for PCOS.

**Methods:**

A randomised controlled, single-blind trial, was carried out from October 2018 to March 2019, which included 122 participants recruited from gynecology outpatient clinics of affiliated Hospital of Zunyi Medical University in Guizhou. The study participants were randomised into intervention (n = 61) and control groups (n = 61). Participants in the intervention group undertook a TTM-based mobile health application program in addition to routine care, and participants in the control group received only routine care.

**Results:**

Fifty-one participants in the intervention group and 49 in the control group completed the study. Compared to the control group, participants in the intervention group showed statistically significant decrease for BMI (P < 0.05), WC (P < 0.05), SAS (P < 0.05), and SDS (P < 0.05) scores at 6-month and 12-month, respectively. Behavior stage change of exercise and diet among paticipants with PCOS was significant at 6 months (c^2^ = 43.032, P < 0.05) and 12th months (c^2^ = 49.574, P < 0.05) between the intervention and control groups.

**Conclusions:**

This study showed that the TTM-based mobile health application program can decrease BMI, WC, anxiety, and depression, and improve exercise and diet adherence in patients with PCOS in the long term. The TTM-based mobile health application program can be applied for lifestyle modification in women with PCOS.

*Trial registration* This study was approved by the ethics committee NO.[2019]1-028 in March 2018 and was registered at the Chinese Clinical Trial Registry (website: www.chictr.org.cn, registry number: ChiCTR2000034572)

## Plain Language summary

Poor adherence of life management in patients with PCOS and many then go on to adherence poor health outcomes as a result. There is an urgent need for new approach to enhance to life management compliance for patients with PCOS. TTM is a common behavior change model, which can effectively promote the behavior change of patients, but it has not been used in the life management of PCOS patients. Meanwhile, the traditional life management of PCOS is mainly face-to-face intervention, few studies have investigated the mobile technology effects on lifestyle modification in PCOS. This study aimed to evaluate the long-term effects of TTM-based mobile technology (for lifestyle modification) on the maintenance of health-related behavior changes among women with PCOS. This study confirmed that the TTM-based mobile health application program can decrease BMI, WC, anxiety, and depression, and improve exercise and diet adherence in patients with PCOS in the long term. Our study conducted TTM-based mobile health application program can provide a reference for other researchers or research organizations.

## Introduction

Polycystic ovary syndrome (PCOS) is the most common endocrine disorder among women of reproductive age; its prevalence varies from 5 to 20% [1, 2]. Women with PCOS experience a variety of symptoms including polycystic ovaries, hyperandrogenism, and menstrual disturbances; more than 80% of women with PCOS are obese or overweight [3, 4]. Women with PCOS might experience complications associated with their reproductive system in addition to a range of metabolic and psychological consequences (depression, anxiety and lower health-related quality of life) [5], including increased risk of type 2 diabetes (T2D), glucose intolerance, and metabolic syndrome [6]. These physiological symptoms positively associated with anxiety and depression are negatively associated with quality of life [7–9]. Evidence-based guidelines recommend lifestyle modification interventions (including, diet, exercise, and behavioral changes) to be the first-line treatment for PCOS, irrespective of the presenting symptoms [10]. Several studies have demonstrated the short-term effectiveness of face-to-face lifestyle modification interventions [11, 12]. However, poor adherence to lifestyle modification remains a concern, especially in the long term [13, 14].

The transtheoretical model (TTM) (also known as the Stages of Change Theory) was initially developed on the basis of findings about individuals who smoke [15]. This model suggests that behavioral change is a dynamic process, comprising the following five stages [16]: (1) pre-contemplation, which refers to the intention to take action in the following 6 months; (2) contemplation, which refers to the intention to engage in healthy behaviors within the following 6 months; (3) preparation, which refers to the readiness to take action within 1 month; (4) action, which refers to behavior change over the past 6 months; and (5) maintenance; which refers to the sustenance of the behavior change for more than 6 months. Several theories have been used to formulate lifestyle modification interventions, focusing on adherence to behavior change; these theories include the Self-Efficacy Theory, Planned Behavior Theory, and Social Learning Theory. However, intervention studies have mostly used the TTM to improve lifestyle modification adherence owing to its effectiveness in the formulation of intervention strategies corresponding to individual characteristics [17]. Several studies have demonstrated the positive effects of the TTM in the promotion and maintenance of health-related behavior changes among patients with knee osteoarthritis, T2D, obesity, ostomy, etc. [17–19]. However, to the best of our knowledge, few studies have used the TTM to develop and carry out interventions for women with PCOS.

Additionally, it is important to note that smartphone ownership rates in China are considerably high; market research conducted in 2021 estimated that there were 1.07 billion smartphone users in China with the highest proportion of netizens being between the ages of 20 and 39 years [20]. This exponential increase in mobile phone usage in developing countries has been accompanied by rapid developments in mobile health technology. Moreover, studies have suggested that the use of mobile technology for health promotion might be effective in improving long-term health-related outcomes [21, 22]. Mobile technology is a promising tool for intervention pertaining to non-communicable diseases in low-resource settings and developing countries [23–25]. Providentially, mobile transfer of health-related information is an action item in the World Health Organization’s Global Action Plan (2013–2020) for developing countries [26].

As women with PCOS experience symptoms at a young age, there is potential to test the feasibility and effectiveness of mobile health applications for improving health-related behavior among these women. However, limited research has been conducted on the same; to the best of our knowledge, only two studies so far have focused on the design of a personalized mobile tool called *AskPCOS*. Research indicates that currently available applications are unlikely to meet their information needs. Additionally, no study has investigated the effectiveness of mobile technology in the interventions for PCOS [27, 28].

In light of the aforementioned arguments, it is necessary to investigate the effectiveness of TTM-based mobile technology for lifestyle modification among women with PCOS. Thus, this study aimed to evaluate the long-term effects of TTM-based mobile technology (for lifestyle modification) on the maintenance of health-related behavior changes among women with PCOS.

## Materials and methods

### Design, setting, and participants

This study was performed using a single-blind randomized controlled trial. Participants were recruited from outpatient gynecology clinics—of the hospital affiliated with Zunyi Medical University in Zunyi—from October 2018 to March 2019. Zunyi is the second largest city in the Guizhou province of China with a population of 6.3 million people, covering an area of 30,762 km^2^. It is located in the neighborhood of Luzhou (in the Sichuan Province) and bordered by Chongqing in the north.

Participants included in the study (1) fulfilled at least two of the three Rotterdam Criteria for the diagnosis of PCOS (including oligo-ovulation or anovulation, hyperandrogenism, and polycystic ovaries that were confirmed via ultrasound) [29]; (2) were aged 18 years or above and had a BMI that was greater than or equal to 25 kg/m^2^; (3) had access to a smartphone; and (4) had a sedentary lifestyle. Participants who were pregnant, had severe disabilities which prevented them from taking care of themselves, or served as participants in other intervention programs for PCOS were excluded from the study.

### Sample size and randomization

A total of 122 participants (61 participants per group) were recruited before the intervention according to inclusion and exclusion criteria. The sample size adequately met the parametric test assumptions, yielding an impact size of 0.30, a power of 90% with 5% margin of error, and allowing an attrition rate of 10%.

An independent researcher prepared the randomization sequence on the basis of a computer-generated block randomization list for 1:1 allocation of participants into two groups (intervention and control groups) with a block size of four. Following the baseline data collection process, participants were randomly assigned to the groups by a researcher who was blinded to the study design.

### Ethical considerations

This study was approved by the institutional review board (IRB) of the hospital affiliated with Zunyi Medical University (Approval no.: 2019/1-028) prior to the commencement of data collection. All participants provided written informed consent for their participation in the study. Furthermore, no adverse events occurred.

### Procedures

Gynecologists identified potentially eligible participants who visited the outpatient gynecology clinics. If the participants fulfilled the inclusion criteria, they were acquainted with the purpose, procedures, and benefits of the study. Data were then collected from participants who provided written informed consent for their participation in the study. Following the baseline data collection process, participants in the intervention group were trained by the researchers to use the mobile health application. Participants attended the outpatient clinic for standardized screening, which was conducted by two researchers who were blinded to group allocation; the measurement time points were as follows: baseline, 6 months, and 12 months. The differences in outcome measures between the intervention and control groups were evaluated across the measurement time points to check for the long-term effectiveness of the TTM-based mobile health application.

#### Intervention group

The participants in the intervention group were required to use the TTM-based mobile health application called *Home of PCOS*, comprising two modules. A multi-disciplinary team—including an obstetrics and gynecology professor, a nursing science professor, a nutritionist, a psychologist, and a software engineer—designed the application. The application was published in collaboration with a software company.

The first module—titled ‘*Assessment of my current behavioral stage*’—of the application required participants to answer a question pertaining to their dietary behaviors and exercise by selecting one of the three alternatives. Based on their responses, participants were divided into the pre-action, action, and maintenance subgroups. Participants were re-assessed for their stage of behavior change every month; accordingly, they were assigned to the relevant subgroup. The second module—titled ‘*What I need to do And how?*’*—*comprised three separate links for the pre-action, action, and maintenance subgroups. Participants were assigned a link based on the assessment of their current behavioral stage; the link comprised specific interventions for the participants.

Pre-action subgroup: This intervention subgroup included three steps (or pages). The first page*—*titled *Knowledge and information about PCOS*—contained recorded lectures and resource pages. The second page*—*titled ‘*How to prepare for lifestyle change?*’—was the provision of knowledge and information to the participants who were preparing for lifestyle changes. The third page—titled ‘*You need to change your lifestyle*’—was an online chat room.

Action subgroup: This intervention subgroup also included three steps (or pages). The first page—titled ‘*Self-monitoring*’—required participants to record their exercise and diet. The second page—titled ‘*Peer support*’—was an online chat room. The third page—titled ‘*Expert consultation*’—was an online chat room.

Maintenance subgroup: This intervention subgroup included two steps (or pages). The first page—titled ‘*Keep up your adherence to lifestyle modification’*—Provided knowledge and information. The second page—titled ‘*Expert consultation*’.—was an online chat room. Table 1 shows a detail description of all intervention components.

**Table 1:** Transtheoretical model-based mobile health application intervention program for polycystic ovary syndrome

Stage of change	Intervention module	Detail strategy
Pre-action (three steps)	① Knowledge and information about PCOS	Contained recorded lectures and resource pages (such as ‘Basic knowledge about PCOS’ and ‘The benefits of a healthy lifestyle for patients with PCOS’) that provided knowledge and information about PCOS
	② How to prepare for lifestyle change	Provided of knowledge and information to the participants who were preparing for lifestyle changes. It contained eight recorded lectures on topics such as home-based exercise programs (including exercise types, duration, and timings), diet and nutrition in PCOS, eating patterns (focusing on the importance of frequency and regularity), and the development of suitable exercise and diet plans
	③ You need to change your lifestyle	In an online chat room: enable one-on-one communication between each participant and the professionally trained researcher, a couple of times;the researcher applied the motivation interview technology to trigger participants’ motivation for modifying their lifestyle
Action (three steps)	① Self-monitoring	Required participants to record their exercise routine (including exercise type, duration, time, and intensity) per week and diet (including daily meals and daily energy intake) per day
	② Peer support	Included a chat room where participants could share their exercise and diet-control experiences with each other
	③ Expert consultation	Included a chat room where participants could consult experts and seek strategies to overcome barriers to lifestyle modification
Maintain (two steps)	① Keep up your adherence to lifestyle modification	Provided knowledge and information to help participants in sustaining their adherence to lifestyle modification
	② Expert consultation	Included a chat room where participants could consult experts and seek strategies to overcome barriers to lifestyle modification

#### Control group

Participants in the control group received routine care; that is, during their visits to the outpatient gynecology clinics, gynecologists gave them advice regarding the maintenance of physical activity and well-balanced diet. The gynecologist provided the same advice to participants in the intervention group.

### Outcome measures

#### Sociodemographic characteristics

Demographic characteristics were measured using the personal information form, which was developed by the researcher and comprised seven questions pertaining to gender, ethnicity, education level, living residence, marital status, occupation, and years of PCOS.

#### Body mass index

The researchers used an electronic scale to measure the participants’ weight. Participants’ height was measured as the length from the top of their head to their sole while they stood in the Frankfort plane (that is, the back of the skull, shoulders, pelvis, and heels touched the same horizontal plane when they stood at attention). It was ensured that the participants were assessed barefoot and in light clothes. The body mass index **(**BMI) was calculated as weight/height^2^ (kg/m^2^) [30].

#### Waist circumference

Waist circumference (WC) of the individuals was measured over their underwear—after a mild expiration while standing—by placing the measuring tape between the edge of the lower costal margin and iliac crest [30]. This measurement was conducted twice per participant and the mean of the two values was used for data analysis.

#### Anxiety

Anxiety was measured using Zung’s Self-Rating Anxiety Scale (SAS), which comprises 20 items that are rated on a 4-point Likert scale, ranging from 1 (“none or a little of the time”) to 4 (“most or all of the time”); total scale scores range from 20 to 80. Cronbach’s alpha for the SAS was 0.82 [31]. Zung recommended the conversion of raw scores to index scores (ranging from 25 to 100) by multiplying the raw score with 1.25. The SAS scores were correlated with the Taylor Manifest Anxiety Scale scores (r = 0.30) [32]. Thus, the SAS is a psychometrically sound measure of anxiety with good sensitivity, specificity, and the capacity to discriminate between anxiety and other psychiatric disorders [2].

#### Depression

Depression status was assessed using Zung’s Self-Rating Depression Scale (SDS), which comprises 20 items that are rated on a 4-point Likert scale, ranging from 1 (“none or a little of the time”) to 4 (“most or all of the time”); total scale scores range from 20 to 80. However, Zung [33] recommended the conversion of raw scores to index scores (ranging from 25 to 100) by multiplying the raw score with 1.25.

#### Behavior change stage

The behavior change stage for exercise and diet was assessed using the following question: “Are you exercising at least thrice a week (for 150 min per week) at a moderate level and maintaining dietary restraint everyday (for example, consuming a low-fat diet)?” Participants responded by choosing one of the following alternatives, which determined the behavior change stage they were in: (a) No, I have never done this (pre-action stage); (b) Yes, I am; however, I have been doing this for less than 6 months (action stage); and (c) Yes, I have been doing this for more than 6 months (maintenance stage).

### Statistical analysis

The SPSS Software (version 18.0) was used for data analysis. Normally distributed data for continuous variables were analyzed using means and standard deviations; whereas, non-normally distributed data were expressed as percentages. Independent sample t-tests were conducted to analyze the statistical significance of the differences in the means of continuous variables between the intervention and control groups. A repeated measures ANOVA was conducted to determine the statistical significance of the effects of the intervention within and between the groups. The Mann–Whitney U and Chi-square tests were conducted to evaluate the statistical significance of differences in the general data (ethnicity, education level, living residence, marital status, occupation, and years of PCOS) between the two groups.

## Results

**Fig. 1:**
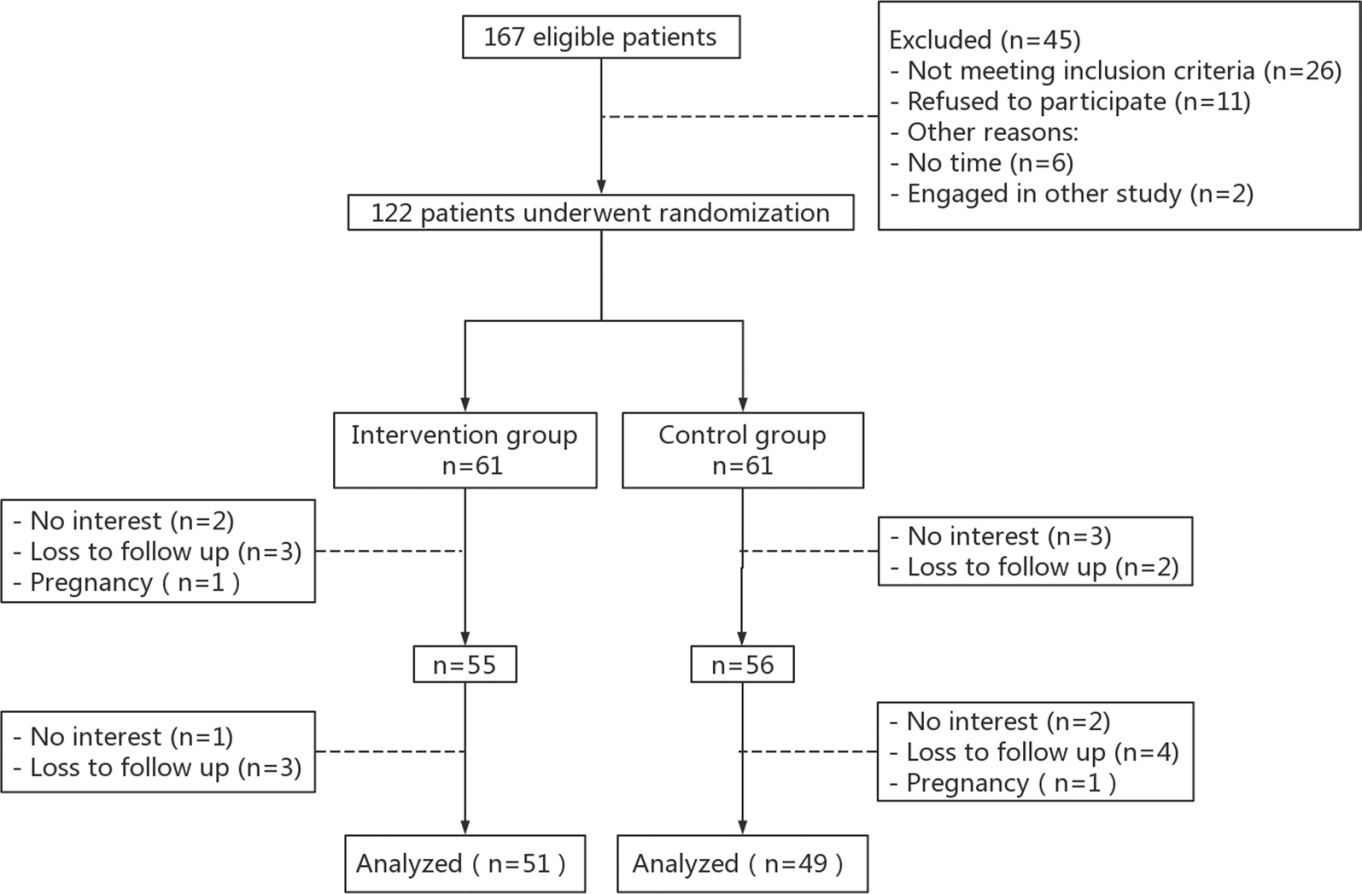
Flow diagram of the progress

Of the 122 participants enrolled in the trial between February 2020 and November 2021, 100 participants completed the study with 51 participants in the intervention group and 49 participants in the control group. Figure 1 shows the participant flow throughout the study. There were no statistically significant differences in the sociodemographic characteristics between participants in the intervention and the control groups (P > 0.05; Table 2).

**Table 2:** Comparison of sociodemographic characteristics of the recruited participants at baseline

Variable	Categories	Intervention group (N = 51)	Control group (N = 49)	χ^2^/t	P value
Age (`x ± s)		24.72 ± 4.20	24.94 ± 4.31	0.37*	0.71
Ethnic group [N(%)]	Han-nationality	31	27	0.331^▲^	0.565
	Ethnic minority	20	22		
Living residence [N(%)]	City	22	21	0.001^▲^	0.997
	Countryside	29	28		
Marital status [N(%)]	Single	29	27	− 0.205^◒^	0.838
	Married	19	17		
	Widowed/divorced	3	5		
Education [N(%)]	Middle school	10	9	0.365^▲^	0.947
	High school	12	14		
	junior college	5	4		
	College	24	22		
Occupation [N(%)]	Employed	13	14	0.856^▲^	0.836
	Unemployed	12	8		
	Student	18	18		
	Other	8	9		
Years of PCOS [N(%)]	< 1 years	28	26	− 0.38^◒^	0.62
	1–3 years	14	12		
	4–6 years	4	4		
	> 7 years	5	7		

^*^ = t; ▲ = χ^2^; ◒ = Mean-Whitney U

Furthermore, there were no statistically significant differences in the BMI (P = 0.38), WC (P = 0.43), SAS scores (P = 0.223), and SDS scores (P = 0.621) between the intervention and control groups at the baseline (Table 3). However, the group-by-time interaction effects on the BMI, WC, SAS scores, and SDS scores were statistically significant, indicating significant group differences on different occasions (P < 0.05). The BMI of participants in the intervention group significantly decreased from the baseline (M = 25.99, SD = 3.87) to the 12th month (M = 22.63, SD = 1.97). Similarly, the WC of participants in the intervention group significantly decreased from the baseline (M = 86.22, SD = 9.91) to the 12th month (M = 80.50, SD = 6.94). However, there were no statistically significant differences in the BMI and WC at different measurement time points among participants in the control group.

**Table 3:** BMI, WC, SAS and SDS of change at baseline, 6-month and 12-month post-intervention among the PCOS patients

Measured parameter	Baseline	6-month	12-month	F(time*group)	P value
BMI				12.8	0.00**
Intervention group	25.99 ± 3.87	23.40 ± 1.69	22.63 ± 1.97		
Control group	25.25 ± 3.95	25.36 ± 3.06	24.86 ± 2.65		
t-value	0.89	− 3.7	6.55		
P-value	0.38	0.00**	0.00**		
WC				2.44	0.04*
Intervention group	86.22 ± 9.91	81.54 ± 8.54	80.50 ± 6.94		
Control group	87.64 ± 6.59	86.75 ± 5.52	85.44 ± 7.95		
t-value	− 0.79	− 3.44	− 3.14		
P-value	0.43	0.00**	0.00**		
SAS				0.91	0.016*
Intervention group	46.97 ± 6.53	42.91 ± 6.65	42.12 ± 9.69		
Control group	49.02 ± 7.05	47.83 ± 6.27	46.46 ± 8.91		
t-value	− 1.23	− 3.49	− 2.14		
P-value	0.223	0.001**	0.035*		
SDS				8.41	0.000***
Intervention group	51.55 ± 6.78	46.83 ± 5.36	40.57 ± 5.41		
Control group	52.29 ± 6.86	50.57 ± 5.42	49.24 ± 7.14		
t-value	0.5	− 2.72	− 6.24		
P-value	0.621	0.008**	0.000***		

F(time * group): The changing trend of measurement indicators in intervention group and control group at different intervention time points

*BMI* body mass index, *WC* Waist circumference, *SAS* Self Rating Anxiety Scale, *SDS* Self Rating Depression Scale

^*^ = P < 0.05;** = P < 0.01:*** = P < 0.001

In addition, the SAS scores of participants in the intervention group significantly decreased from 46.97 (SD = 6.53) at the baseline to 42.12 (SD = 9.69) at the 12th month (P = 0.035). The SDS scores of participants in the intervention group significantly decreased from the baseline (M = 51.55, SD = 6.78) to the 12th month (M = 40.57, SD = 5.41; P < 0.001). In contrast, there were no statistically significant differences in the SAS and SDS scores at different measurement time points among participants in the control group.

**Fig. 2:**
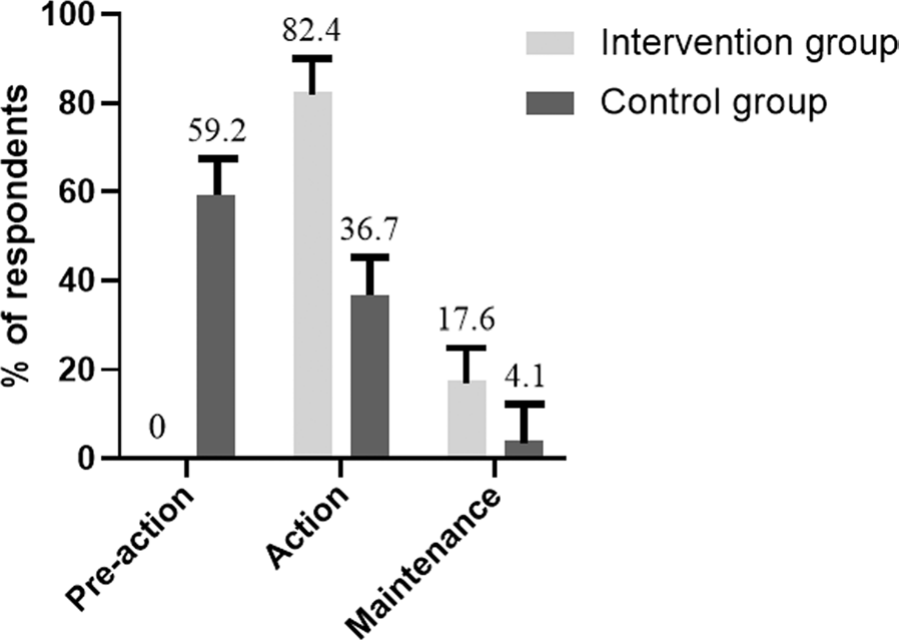
Behavior stage change of exercise and diet among patients with PCOS at the 6th month

There was a significant difference in the behavior change stage for exercise and diet between the intervention and control groups at the 6th month (c^2^ = 43.032, P < 0.05); that is, 82.4% of the participants in the intervention group were in the action stage of behavior change, whereas, there was no significant change in behavior stage from the baseline to the 6th month (P > 0.05) among participants in the control group (Fig. 2).

Furthermore, there was a significant difference in the behavior change stage for exercise and diet between the intervention and control groups at the 12th month (c^2^ = 49.574, P < 0.05). In contrast, there was no significant change in the behavior stage among participants in the control group at the 12th month (P > 0.05). Interestingly, 33.3% of the participants in the intervention group had transitioned to the action stage, and 66.7% of the participants had transitioned to the maintenance stage; whereas only 10.2% of participants in the control group had completed the pre-action and action stages, and entered the maintenance stage.

## Discussion

A review of the extant literature suggests that the findings pertaining to the feasibility and effectiveness of mobile health interventions are inconsistent. A previous study comparing the benefits of of a mobile health application with routine care among people with chronic obstructive pulmonary disease found no statistically significant differences in improving the health-related quality of life; whereas few studies have highlighted the effectiveness of mobile-application based health interventions.

**Fig. 3:**
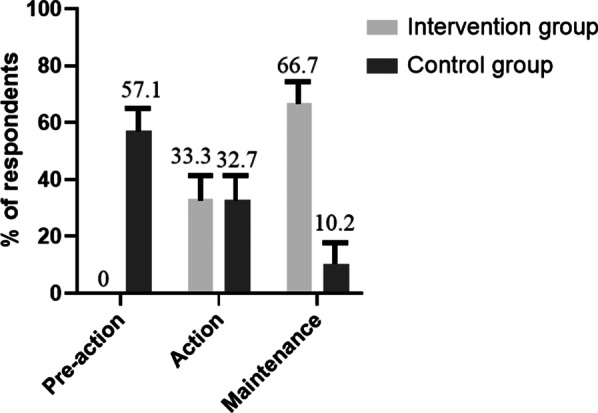
Behavior stage change of exercise and diet among participants with PCOS at the 12th month

The current study was one of the few studies that examined the effectiveness of a TTM-based mobile health intervention program (using the mobile application called *Home of PCOS*) on the maintenance of self-managed health-related behavior change among women with PCOS. BMI and WC are important clinical indices that are associated with PCOS symptoms. A recent systematic review reported that dietary modifications alone were associated with a significant decrease in the BMI [34]. Another systematic review showed a statistically significant decrease in the BMI and WC as a consequence of short-duration (less than 12 weeks) and supervised aerobic exercise-based interventions with or without diet combined [35]. On the contrary, the current study found that the maintenance of self-managed health-related behavior change—including exercise and diet—led to a significant decrease in the BMI and WC over time (at both 6 and 12 months), highlighting the efficacy of the combination of routine care with the use of *Home of PCOS* (intervention group) as opposed to only receiving routine care (control group)*.* These findings could be attributed to the TTM-based mechanisms underlying the mobile health application in the current study that facilitated the initiation and management of behavior change among participants in the intervention group. During the action stage, participants used strategies such as self-monitoring and peer support—through the mobile application—which helped them to maintain health-related behaviors (Fig. 3).

Women with PCOS experience higher levels of anxiety and depressive symptoms as compared to women without PCOS [36, 37], prompting international guidelines to recommend the management of anxiety and depressive symptoms among women with PCOS [38]. Some studies have suggested that lifestyle management could result in significantly lower levels of depression [39, 40]. However, a 20-week long diet and exercise intervention study found that low level of depressive symptoms among participants in the intervention group could only be sustained during the first 10 weeks [41]. Furthermore, two diet-induced weight loss studies also found short-term improvement in psychological outcomes; however, this improvement could not be sustained in the long-term [42, 43]. Nevertheless, the current study found significant improvements in anxiety and depressive symptoms among participants in the intervention group at 6 months; the improvements were sustained at 12 months. The aforementioned findings can be attributed to the following factors. First, significant weight loss among women with PCOS has been found to have positive effects on psychological outcomes [44]. Second, lack of information for the management of PCOS might lead to anxiety and depressive symptoms among women with PCOS [45]. The present study facilitated the provision of PCOS-related information to participants in the intervention group via the mobile health application. This might have had positive implications on the participants’ psychological health.

Maintaining health-related behavior changes in the long term is challenging for women with PCOS. Many traditional face-to-face interventions have reported poor adherence to lifestyle modification. A systematic review reported [6] that only 50% of PCOS patients persisted in lifestyle modification during lifestyle intervention in the short term (less than 12 months). Another study involving a 12-month lifestyle intervention for women with PCOS reported 54% adherence to lifestyle modification [46]. To the best of our knowledge, this is the first study to have investigated the long-term effectiveness of a TTM-based mobile health application on health-related behavior changes (including exercise and diet) among patients with PCOS. The positive effects of the TTM-based mobile health application on the stages of behavior change could be attributed to several factors. First, the ingression to the action stage primarily depends on their knowledge, skills, and motivation. The TTM-based mobile health application provided knowledge, and strategies for lifestyle modification, in addition to motivating the participants to adhere to their lifestyle changes. Second, most people who take action to change their behaviors are not successful in maintaining it in the long term [47]. In the current study, participants were assessed for their behavior change stage for diet and exercise every month; accordingly, the mobile health application provided corresponding intervention strategies. The intervention strategies for improving health-related behavior were flexible; this could have prevented the regression to the previous behavior change stages. Third, mental health outcomes such as depression and anxiety negatively influence trial adherence [48]. In this study, we found significant improvements in anxiety and depressive symptoms at 6 months among participants in the intervention group; these improvements were sustained at 12 months. This might have positively influenced the adherence to healthy behaviors.

It is important to acknowledge the limitations of this study. First, participants were recruited from one general hospital; this might have limited the generalizability of the findings. Second, the study assessed self-reported adherence to the health-related behavior changes; these changes were not monitored by the researchers. Furthermore, recall bias could have reduced the accuracy of the findings. Third, the time span for follow-up regarding health-related behavior change was short (12 months). Future studies with longer duration of intervention, and carry out more TTM-based mobile health applications multicenter RCT research are required to confirm the validity of our findings.

## Conclusion

This study showed that the TTM-based mobile health application—*Home of PCOS*—had significant long-term effectiveness in reducing BMI, WC, anxiety, and depressive symptoms and improving adherence to health-related behavior changes among women with PCOS. The application can be used in addition to the provision of routine care to facilitate adherence to lifestyle modification among women with PCOS.

## Data Availability

The dataset used during the current study is available from the corresponding author on reasonable request.

## References

[1] Broekmans FJ, Knauff EAH, Valkenburg O, Laven JS, Eijkemans MJ, Fauser BCJM (2006). PCOS according to the Rotterdam consensus criteria: change in prevalence among WHO-II anovulation and association with metabolic factors. BJOG.

[2] Bozdag G, Mumusoglu S, Zengin D, Karabulut E, Yildiz BO (2016). The prevalence and phenotypic features of polycystic ovary syndrome: a systematic review and meta-analysis. Hum Reprod.

[3] The Rotterdam ESHRE/ASRM-Sponsored PCOS Consensus Workshop Group (2004). Revised 2003 consensus on diagnostic criteria and long-term health risks related to polycystic ovary syndrome. Fertil Steril.

[4] Fauser BC, Tarlatzis BC, Rebar RW, Legro RS (2012). Consensus on women's health aspects of polycystic ovary syndrome (PCOS): the Amsterdam ESHRE/ASRM-Sponsored 3rd PCOS Consensus Workshop Group. Fertil Steril.

[5] Banting LK, Gibson-Helm M, Polman R, Teede HJ, Stepto NK (2014). Physical activity and mental health in women with polycystic ovary syndrome. BMC Womens Health.

[6] Moran LJ, Misso ML, Wild RA, Norman RJ (2010). Impaired glucose toler- ance, type 2 diabetes and metabolic syndrome in polycystic ovary syndrome: a systematic review and meta-analysis. Hum Reprod Update.

[7] Kakoly NS, Khomami MB, Joham AE, Cooray SD, Misso ML, Norman RJ (2018). Ethnicity, obesity and the prevalence of impaired glucose tolerance and type 2 diabetes in PCOS: a systematic review and meta-regression. Hum Reprod Update.

[8] Kaczmarek C, Haller DM, Yaron M (2016). Health-related quality of life in adolescents and young adults with polycystic ovary syndrome: a systematic review. J Pediatr Adolesc Gynecol.

[9] Nasiri Amiri F, Ramezani Tehrani F, Simbar M, Montazeri A, Mohammadpour Thamtan RA (2014). The experience of women affected by polycystic ovary syndrome: a qualitative study from iran. Int J Endocrinol Metab.

[10] Teede HJ, Misso ML, Costello MF, Dokras A, Laven J, Moran L (2018). Recommendations from the international evidence-based guideline for the assessment and management of polycystic ovary syndrome. Hum Reprod.

[11] Benham JL, Yamamoto JM, Friedenreich CM, Rabi DM, Sigal RJ (2018). Role of exercise training in polycystic ovary syndrome: a systematic review and meta-analysis. Clin Obes.

[12] Kite C, Lahart IM, Afzal I, Broom DR, Randeva H, Kyrou I, Brown JE (2019). Exercise, or exercise and diet for the management of polycystic ovary syndrome: a systematic review and meta-analysis. Syst Rev.

[13] Moran LJ, Hutchison SK, Norman RJ, Teede HJ. Lifestyle changes in women with polycystic ovary syndrome. Cochrane Database Syst Rev. 2011:CD007506. 10.1002/14651858.CD007506.pub2 21328294

[14] Moran LJ, Noakes M, Clifton PM, Tomlinson L, Galletly C, Norman RJ (2003). Dietary composition in restoring reproductive and metabolic physiology in overweight women with polycystic ovary syndrome. J Clin Endocrinol Metab.

[15] Leer EV, Hapner ER, Connor NP (2008). Transtheoretical model of health behavior change applied to voice therapy. J Voice.

[16] Prochaska JO, Diclemente CC (1982). Transtheoretical therapy: toward a more integrative model of change. Psychother Theory.

[17] Wang L, Chen H, Lu H (2020). The effect of transtheoretical model-lead intervention for knee osteoarthritis in older adults: a cluster randomized trial. Arthritis Res Ther.

[18] Alime S (2019). The effect of a transtheoretical model-based motivational interview on self-efficacy, metabolic control, and health behaviour in adults with type 2 diabetes mellitus: a randomized controlled trial. Int J Nurs Pract.

[19] Wen S-L, Li J, Wang A-N (2018). Effects of transtheoretical model-based intervention on the self of patients with an ostomy: a randomised controlled trial. J Clin Nurs.

[20] Xie J, Frada B, Rhonda G (2018). Personalized mobile tool AskPCOS delivering evidence-based quality information about polycystic ovary syndrome. Semin Reprod Med.

[21] Miller R, Qiang CZ, Yamamichi M (2012). Mobile applications for the health sector.

[22] Wang L, Guo Y, Wang M, Zhao Y (2021). A mobile health application to support self-management in patients with chronic obstructive pulmonary disease: a randomised controlled trial. Clin Rehabil.

[23] Bloomfield GS, Vedanthan R, Vasudevan L, Kithei A, Were M, Velazquez EJ (2014). Mobile health for non-communicable diseases in Sub-Saharan Africa: a systematic review of the literature and strategic framework for research. Glob Health.

[24] Aranda-Jan CB, Mohutsiwa-Dibe N, Loukanova S (2014). Systematic review on what works, what does not work and why of implementation of mobile health (mHealth) projects in Africa. BMC Public Health.

[25] Déglise C, Suggs LS, Odermatt P (2012). SMS for disease control in developing countries: a systematic review of mobile health applications. J Telemed Telecare.

[26] WHO (2012). World Health Organizations zero draft: global action plan for the prevention and control of noncommunicable diseases 2013–2020.

[27] The 48th Statistical report on internet development in China [EB/OL]. https://news.cctv.com/2021/08/27ARTIAQ8bIAmQ68Vs88OMHnRa210827.shtml.

[28] Jacqueline B, Xu R, Emily G (2018). Ask PCOS: identifying need to inform evidence-based app development for polycystic ovary syndrome. Semin Reprod Med.

[29] Rotterdam EA-SPCWG (2004). Revised 2003 consensus on diagnostic criteria and long-term health risks related to polycystic ovary syndrome. Fertil Steril.

[30] Pekcan G. In: Baysal A, Aksoy M, Besler HJ, editors. ve arkBeslenme Durumunun Saptanmasi. İçinde: Diyet El kitabı. Yenilenmis 6. Baski. Ankara: Hatipoglu Yayinlari. 2011. pp. 67–143. **(In Turkish)**.

[31] Tanaka-Matsumi J, Kameoka VA (1986). Reliabilities and concurrent validities of popular self-report measures of depression, anxiety, and social desirability. J Consult Clin Psychol.

[32] Zung WWK (1971). A rating instrument for anxiety disorders. Psychosomatics.

[33] Zung WWK (1965). A self-rating depression scale. Arch Gen Psychiatry.

[34] Abdolahian S, Tehrani FR, Amiri M (2020). Effect of lifestyle modifications on anthropometric, clinical, and biochemical parameters in adolescent girls with polycystic ovary syndrome: a systematic review and meta-analysis. BMC Endocr Disord.

[35] Kite C, Lahart IM, Afzal I (2019). Exercise, or exercise and diet for the management of polycystic ovary syndrome: a systematic review and meta-analysis. Syst Rev.

[36] Veltman-Verhulst SM, Boivin J, Eijkemans MJ, Fauser BJ (2012). Emotional distress is a common risk in women with polycystic ovary syndrome: a systematic review and meta-analysis of 28 studies. Hum Reprod Update.

[37] Cooney LG, Lee I, Sammel MD, Dokras A (2017). High prevalence of moderate and severe depressive and anxiety symptoms in polycystic ovary syndrome: a systematic review and meta-analysis. Hum Reprod.

[38] Teede HJ, Misso ML, Costello MF, Dokras A, Laven J, Moran L (2018). Recommendations from the inter- national evidence-based guideline for the assessment and management of polycystic ovary syndrome. Fertil Steril.

[39] Clark AM, Thornley B, Tomlinson L, Galletley C, Norman RJ (1998). Weight loss in obese infertile women results in improvement in reproductive outcome for all forms of fertility treatment. Hum Reprod.

[40] Clark AM, Ledger W, Galletly C, Tomlinson L, Blaney F, Wang X (1995). Weight loss results in significant improvement in pregnancy and ovulation rates in anovulatory obese women. Hum Reprod.

[41] Thomson RL, Buckley JD, Lim SS, Noakes M, Clifton PM, Norman RJ (2010). Lifestyle management improves quality of life and depression in overweight and obese women with polycystic ovary syndrome. Fertil Steril.

[42] Bacon L, Stern JS, Van Loan MD, Keim NL (2005). Size ac- ceptance and intuitive eating improve health for obese, female chronic dieters. J Am Diet Assoc.

[43] Karlsson J, Hallgren P, Kral J, Lindroos A-K, Sjöström L, Sullivan M (1994). Predictors and effects of long-term dieting on mental well-being and weight loss in obese women. Appetite.

[44] Naderpoor N, Shorakae S, de Courten B, Misso ML, Moran LJ, Teede HJ (2015). Metformin and lifestyle modification in polycystic ovary syndrome: systematic review and meta-analysis. Hum Reprod Update.

[45] Ee C, Smith C, Moran L, MacMillan F, Costello M, Baylock B, Teede H (2020). "The whole package deal": experiences of overweight/obese women living with polycystic ovary syndrome. BMC Womens Health.

[46] Hoeger KM, Kochman L, Wixom N, Craig K, Miller RK, Guzick DS (2004). A randomized, 48-week, placebo-controlled trial of intensive lifestyle modification and/or metformin therapy in overweight women with polycystic ovary syndrome: a pilot study. Fertil Steril.

[47] Prochaska JO (1984). Systems of psychotherapy: a transtheoretical analysis.

[48] Pastore LM, Patrie JT, Morris WL, Dalal P, Bray MJ (2011). Depression symptoms and body dissatisfaction association among polycystic ovary syndrome women. J Psychosom Res.

